# Impact of individual and environmental factors on academic performance of pregnant adolescent

**DOI:** 10.1186/s12905-023-02520-y

**Published:** 2023-07-21

**Authors:** Bolajoko Elizabeth Otegbayo, Noralina Omar, Mahmoud Danaee, Samira Mohajer, Nasrin Aghamohamadi

**Affiliations:** 1 Centre for Epidemiology and Evidence-Based Practice, Department of Social and Preventive Medicine, Faculty of Medicine, University of Malaya, Kuala Lumpur, 50603 Malaysia; 2 Institute for Advanced Studies, University of Malaya, Kuala Lumpur, 50603 Malaysia; 3 Faculty of Arts and Social Sciences, Department of Social Administration and Justice, University of Malaya, Kuala Lumpur, 50603 Malaysia; 4 Nursing and Midwifery Care Research Centre, Mashhad University of Medical Sciences, Mashhad, Iran

**Keywords:** Adolescent pregnancy, Academic performance, Environment, Sexual attitude, Urban health

## Abstract

**Background:**

Teenage pregnancies continue to disrupt teenage girls’ academic development. As a result, teenage mothers are at risk of unemployment, maternal death, and poverty. Previous research, however, has shown that both individual and environmental factors can have a significant impact on the prevalence of adolescent pregnancy. However, there has been little rigorous research on the impact of these factors on pregnant students’ academic performance.

**Objectives:**

The purpose of this study was to determine the relationship between environmental (neighbourhood) and individuals (sexual attitudes, peer attachment) factors. It also examined the influence of individual factors on the academic performance of pregnant teens.

**Methods:**

The study included a cross-sectional study of 400 pregnant adolescent students aged 15–19 years. The target groups were drawn from three major cities in Nigeria. Respondents were identified through targeted snowballing. Pregnant participants were a combination of married and unmarried girls attending school from home. Data were collected using a structured and self-completed questionnaire. Thus, frequency, mean and standard deviation were used for descriptive analysis. Pearson correlation analysis was applied to show the relationship between variables.

**Results:**

The study found that neighbourhood (r=-.125, p = .12) had a negative and significant relationship with peer attachment. However, there was no significant evidence of a relationship between sexual attitudes and neighbourhood (r=-.040, p = .422). There was, however, a significant relationship between sexual attitudes and academic performance (r = .236, p = .000). There was also a relationship between peer attachment and academic performance (r=-.401, p = < 0.001).

**Conclusion:**

This study suggests that the academic performance of pregnant teenagers necessitates a combination of approaches, which includes changes in personal and prosocial behaviour, and environmental reforms. This can be achieved through, peer education, school day-care, subsidised or free contraceptives, free or subsidized education, and community programmes that positively influence young adults in the neighbourhood. These approaches can indirectly boost self-efficacy, motivation, and confidence to achieve higher academic feat, while reducing school dropout rate among the target groups.

## Introduction

Teenage pregnancies are a public health challenge in both developed and developing countries. According to global records, nearly 21 million teenage girls become pregnant each year, 10 million of them unintentionally [1]. Sub-Saharan Africa accounts for 95% of the world’s adolescent population [2]. Nigeria is one of the countries with the highest incidence of teenage pregnancy in sub-Saharan Africa and West Africa [3]. An estimated 45% of girls aged 15–19 are sexually active[3] and 23% of this group already have a child [4]. In addition, 1.3 million adolescent girls in Nigeria drop out of school every year [5]. A significant 7.3 million girls are excluded from access to education in Nigeria each year [6].

Some of the causes of teenage pregnancies in Nigeria are limited access to sexual health services, low knowledge of contraceptives and their use, unequal distribution of health infrastructure and services, and inadequate knowledge about sexual and reproductive health [7–9]. Teenage pregnancies are considered a leading cause of maternal and child deaths, school dropout, unemployment, and poverty among teenage mothers worldwide [1, 10, 11]. Studies have linked neighbourhood poverty, negative peer influence, weak parental control and relationships, lack of sexual health knowledge and risky sexual practices to causes of unwanted pregnancies [12, 13].

For example,Brahmbhatt et al. found that living in a poor residential environment, where access to basic amenities was a luxury, encouraged negative peer influence leading to early sexual debut among respondents. Socio-economic status was identified as an indicator of poverty in the neighbourhood. This makes teenage girls vulnerable to early social independence and may promote negative peer pressure [14]. The lack of sexual health facilities and recreational activities or community tasks to engage teenagers in the environment also exacerbates the consequences of risky sexual behaviour and increases the risk of unwanted pregnancies [14–16].

Pregnant students’ decisions to continue or discontinue their studies may depend on their academic performance [17]. Like all other adolescents, pregnant female students face the same challenges as other students because they are students first and expectant mothers second. Unfortunately, pregnant female students are treated with hostility, stigma, and ridicule by both teachers and their peers, which reduces their motivation to learn [18]. These and other difficulties increase the likelihood of school dropout among this target group. It is likely that the lack of adequate social and environmental support for pregnant teens may negatively affect their academic success.

Undoubtedly, getting pregnant as a teenager is difficult; therefore, the combination of pregnancy and school and lack of support can exacerbate the experience and discourage female teens from continuing their education. And because the United Nations considers education a basic human right, the lack of support for academic achievement or improvement can be seen as a violation of that right [5]. In addition, young girls and the female population continue to be denied their rights to social and economic prospects due to teenage pregnancies [5].

This has consistently exposed women to the dangers of domestic violence, lack of sexual autonomy, child marriage, unemployment, and poverty [6, 19]. In Nigeria, teen pregnancy-related school drop-out rates continue to increase the illiteracy rate of women [20, 21]. Despite the enormous resources devoted to preventing teen pregnancy, more females become pregnant each year. Which begs the question, “Should teenage pregnancy continue to deny females their fundamental right to education?“ Noting that if the causes of this pregnancy are not addressed, it may be more difficult for them to achieve academic success.

This supports the need to investigate factors that may affect the academic performance of pregnant adolescent students. These students can be motivated to pursue academic success by exerting effort in this area. Furthermore, this can improve the social and economic prospects of adolescent mothers and their children. Considering this, research has identified several variables, including peer attachment and sexual attitudes, as predictors of academic progress among expectant adolescents. Girls experience rapid physical, cognitive, and psychological development during their adolescent years [22, 23]. These abrupt changes frequently impair their capacity for reasoning, feeling, interacting, and making decisions [24, 25]. This makes them more likely to engage in sexual activity or seek external validation from peers [24, 25]. The trajectory of their peer associations can be a major determinant of how they respond to sexual health issues and their academic engagement [24, 25].

Current studies examining the effects of adolescent sexual activity on educational outcomes primarily argue that early romantic relationships are time-consuming and may impose significant emotional costs on adolescents, particularly if they are female [24, 26, 27]. Evidence from studies suggests that, sexually active adolescents’ girls are significantly less likely to invest in their own education [24, 26, 27].

Early sexual debut among adolescents in secondary school is considered a predictor of academic achievement. Frisco believes that early sexual debut can influence dropping out of the academic year or even hinder advancement to earn a diploma following post-secondary education [24]. The author linked unsafe sexual activity such as the lack of contraceptive measures at first sexual debut and teenage motherhood status to this claim [24].

Consistently Ventrina, also buttressed that sexual heterosexual engagement and non-use of contraceptive equally among adolescent students significantly predicted academic performance [28]. According to Ventrina sexual active students struggled to balance sexual engagement with academic concentration, and this affected their commitments towards their academics [28]. In addition, Kumar confirmed that adolescent students with average academic performance did not engage in any form of sexual activity, while students with poor academic performance engaged in sexual activity [26]. This suggests importance of sexual attitude on academic performance. Sexual attitude especially in case of sexual engagement and non-use of contraceptives can predispose teenagers to academic risks by reducing their motivation to learn, as well as their feeling of connectedness to academic performance.

Peer attachment is one of the most important determinants of academic achievement because puberty is a time when adolescents seek validation and acceptance from peers[25, 29, 30]. Therefore, the quality of peer relationships is critical, as it can determine success or failure. Thus, interactions with peers can be characterised as positive or negative, and both directions can determine to some degree the influence on adolescents’ academic performance [31]. An adolescent girl who maintains a circle of friends with negative influence who engage in risky sexual health and behaviours such as truancy, substance abuse, and unprotected sex is likely to experience peer pressure because she is seeking validation and acceptance from peers [25].

Maintaining such a circle of friends can reduce self-motivation to attend school or learn well, which in turn can affect academic performance[25]. In comparison, Liu et al. found in their study that students’ academic achievement was moderated by peer influence, meaning that students who were in a circle of friends with high academic engagement had high academic achievement compared to students who were in the company of a negative influence with low academic achievement [32]. Accordingly, Llorca et al. also found a direct relationship between peer attachment quality and self-efficacy related to positive academic achievement, while low efficacy related to academic achievement was directly related to negative peers [33]. In summary, the role of positive peer attachment in improving teenage girls’ academic achievement is important.

According to human ecology theory, a child’s environment and social interaction can determine their development. For example, the quality of a child’s environment in terms of access to school, a quality and accessible health system, and community activities to engage young adults can predict academic performance, onset of early sexual activity and other health risk behaviours [34–36]. The quality of a teenage girl’s environment, as mentioned earlier, can also determine the nature of her peer relationships, which can lead to negative or positive peer influence, which in turn can affect her attitudes towards sexual activity and academic success [34–36].

The neighbourhood environment plays an important role in shaping the personality and behaviour of the adolescent. The environmentalist believes that no child is born a genius, a slacker, or a criminal [37–39]. It is the environment that decides the outcome of a child. An adolescent girl’s propensity for deviant behaviour (risky sexual behaviour) depends on the type of peers she surrounds and associates within her environment [40, 41]. In an earlier study by Crane [42], school dropout and pregnancy among adolescents were found to be caused by a bad neighbourhood [42]. The study found that a bad neighbourhood predicted peer pressure and that this significantly influenced the acceptability of teenage pregnancy and school dropout among neighbourhood adolescents. In comparison, [43] found that negative peer influence and unhealthy peer relationships significantly influenced teenage sexual initiation, risky sexual behaviour, and pregnancies. However, studies have shown that individual health modifications can alter the negative effect of poor neighbourhood environment, unsafe sexual behaviour, and negative peer influence in adolescent girls. Behavioural control can alter the perceptions of individual and environmental consequences of negative peer influence on unsafe sexual practices. Improved sexual behaviour can foster self-efficacy, self-confidence, and self-esteem and in turn influence positive academic outcome.

Despite numerous studies on the causes and consequences of teenage pregnancy, teenage pregnancies continue to rise and pose a threat to the education and academic integration of pregnant teenage female students. The question arguably remains whether teenage pregnancy should continue to threaten the educational progress of this vulnerable population knowing that this will further increase female illiteracy and make women vulnerable to gender inequality, domestic violence, unemployment, and poverty. It is important to examine the impact of individual and environmental factors on the academic performance of pregnant female students in Nigeria as there is limited evidence on this topic. Studies in this direction can provide new insights that can guide policy makers in developing social intervention strategies for the integration of teenage students into the academic space. The current study examined the relationship between individual and environmental factors and assessed the relationship between individual factors and the academic performance of pregnant female students. The effects of these factors on academic performance can serve as a guide for policymakers and key stakeholders such as governments and non-governmental organisations (NGOs) in developing culturally tailored interventions and policies that will benefit pregnant adolescent students. It can also improve the target group’s social opportunities. Furthermore, the findings may overlap with Sustainable Development Goals 3 and 6, which aim to improve women’s education, equality, and quality of life.

## Methodology

### Research design

The study was a quantitative and cross-sectional study design. A total of 400 consenting pregnant teenagers were enrolled into the study. Data were collected using a structured, self-completed questionnaire.

### Population of study

A total of 400 older adolescent girls aged 15–19 years who were pregnant at the time of data collection participated in the study. The reason for choosing older adolescent girls is that according to Nigeria Health Demographic data, this age group has a high rate of unplanned pregnancy and childbirth in Nigeria. The mean age was 17.25 years (SD = 1.30), and most of the study population was married and only a few were unmarried. The respondents who were married lived with their spouse, while the unmarried lived either with their parents or extended family members (grandparents or uncles and aunts). A significant proportion of respondents lived in urban areas. Further, majority of respondents were a combination of Hausa, Yoruba, Igbo ethic group, and other ethnic groups were sparsely represented in the study as well. All pregnant respondents were enrolled in school and attended from home during the survey.

### Sampling procedure and sampling

Respondents were recruited from the northern (Niger state), southern (Port Harcourt), and western (Lagos) regions of Nigeria within 8–16 weeks (January-April 2022). These regions were selected due to the high prevalence of teenage pregnancy and recommendation gotten from ministry of health Nigeria and some experts in Nigeria. These three (3) regions also represent the diverse cultural heritage of Nigeria. A purposive snowballing method was used to select the study respondents. This method was chosen because the issue of teenage pregnancy is very sensitive in Nigeria. In addition, fear of stigmatization could hinder the identification of participants. Therefore, a referral method was used to recruit pregnant teenagers for the study. The selection criterion for recruitment was pregnancy and age between 15 and 19 years and been enrolled in school. Respondents were approached at various locations (homes, shelter homes, schools, and health centres) within the selected study sites, depending on where the referrals came from. Written informed consent was provided to both the parents/guardians of the participants and the participants themselves prior to data collection. Consenting respondents were given questionnaires and given adequate time to complete the questionnaires.

The initial sample size was 384 and was rounded to 400 to compensate for possible non-response and missing data. The sample size was calculated using the formula of Krejcie & Morgan [44]. The sampling formula for the current study is expressed as shown in Eq. 1:

1 $$S=\frac{{X}^{2} NP (1-p)}{{d}^{2} \left(N-1\right)+{X}^{2} P (1-P)}$$

Where, s = sample size, N = population size, d^2^ = acceptable sampling error, x^2^ = chi-square degree of freedom and P = proportion of population as shown in Eq. 2.

2 $$S=\frac{{3.841}^{*} {\text{22,772}}^{*} {0.5}^{*}0.5}{\begin{array}{c}{\left(\right(0.05)}^{*} \left(\text{22,667,772})\right)+{(3.841}^{*} {0.5}^{*}0.5)\\ \text{n}=384\end{array}}$$

### Research instrument

The items for neighbourhood environment were adopted from Akintayo’s study [45]. Fourteen (14) indicators were used to measure these variables. Attachment to peers was assessed with 12 items. These items were taken from the Inventory of Peer and Parent Attachment (IPPA) [46]. In addition, the items operationalising sexual attitudes were taken from the Assessment of Sexual Knowledge (ASK) [47]. A total of 11 items were used to assess sexual attitudes. For measuring academic performance, 3 items were used, which were adopted from Jesus Montero-Marin et al.[48] and Jesus Montero-Marin et al.[49] To ensure the validity and reliability of the study instrument, the adopted items were reviewed and sent to five experts for validation. The items included in the scale were reviewed by different experts in the field of the topics concerned. A pilot study (test and retest) was conducted with 30 consenting pregnant teenagers. The responses were compared to measure the internal consistency and accuracy of the study instrument. Thus, kappa (0.45–0.677) for sexual attitudes, α = 0.90 for neighbourhood environment and α = 0.52–0.63 for peer attachment were obtained as reliability indices.

### Study variables

**Environmental factors** measured “*Quality of the living environment (Neighbourhood)*” and it was measured by safety of life and property, proximity to school, business and job opportunity, availability of telecommunications, air/noise pollution, availability of sports centres, health care centres, history of flood or mitigation, social opportunities, cleanliness of neighbourhood, and available leisure activities. Each statement on these variables was answered with a 5-point Likert scale ranging from ‘1’ very adequate to ‘5’ very inadequate. While **Individual factors** measured “*Peer attachment*” and *“Sexual attitudes”*. *Peer attachment-* was defined as the degree of attachment, emotional support, openness, and control shown to gain trust and openness to communicate with friends. Therefore, peer attachment was assessed with 12 items. The degree of attachment to peers was rated on a Likert scale, with “not at all” rated as “5” and “very much” rated as “1”. *Sexual attitudes*- was defined as the ability to make informed choices, such as personal responsibility, willingness, and trust in unsafe sexual practises to prevent unwanted pregnancy. These variables had a dichotomous response format. Therefore, for each item, a scale of 1 = yes, 2 = no and 0 = I do not know was evaluated.

Academic performance - this was measured in accordance with the definition and measure of Jesus Montero-Marin et al. and Jesús Montero-Marin et al. Therefore, cumulative grade point average (CGPA) also known as academic records, ability to concentrate during studies, and number of failed subjects were evaluated as measures of academic performance. The answers to the CGPA questions were therefore evaluated with a Likert score of 1 = < 2.5, 2 = 2.5–3.5 and 3 = > 3.5. As a result, factors such the cumulative grade point average (CGPA), the capacity to focus while studying, and the number of subjects failed were assessed. Thus, the answers to the CGPA questions were given a Likert value of 1, 2, or 3, depending on whether they were positive or negative. One of the questions on academic performance was subjective in nature as it related to personal perceptions that determine the ability to concentrate in learning. As a result, responses were graded on a scale of 1 = Always, 2 = Sometimes, and 3 = Never. Additionally, responses to subject failed were graded on a scale of 1 = 2 or more, 2 = 1, and 0 = None.

### Data analysis

The analysis for this study was conducted using SPSS version 23 software. Thus, frequency, mean and standard deviation were used for descriptive analysis. Pearson correlation analysis was applied to show the relationship between the dependent (academic performance) and independent variables (neighbourhood, sexual attitude, and peer attachment).

## Result

### Demographic characteristics of respondents

Table 1 shows the distribution of the demographic variables of the respondents. A significant proportion of pregnant respondents attended either primary school (35%) or secondary school (39.5%), while a moderate proportion attended tertiary education (20.3%). Most respondents were married (63.7%), while a moderate number were single (36.5%). In addition, 64.8% of the respondents were from low-income families and about 26% from middle-income families and a very small percentage of 9.3 from high-income families. Furthermore, the Hausa (45%) ethnic group was found to be the majority population, followed by Yoruba (21%), Igbos (17%), and Others (16.3%).

**Table 1:** Frequency Distribution of Demographic Profile

*No*	Variables	Category	n	%
1.	Level of education	Primary	140	35
		Secondary	158	39.5
		Tertiary	81	20.3
4.	Marital Status	Married	255	63.7
		Single	145	36.5
6.	Household income	Low income	259	64.8
		Middle income	104	26
		High income	37	9.3
9.	Ethnic Group	Hausa Yoruba Igbo Others	182 85 68 65	45.5 21 17.0 16.3

### Relationship between neighbourhood and peer attachment

The first aim of this study was to examine the relationship between the quality of the neighbourhood and pregnant girls’ attachment to peers. The study revealed that the mean score of neighbourhood environment was M = 2.59, SD = 0.64 and frequency analysis showed that 69.3% of the respondent’s lived-in low-quality neighbourhood while 30.8% lived-in high-quality neighbourhood. This indicates that the quality of the respondents’ living environment was poor. The study used Pearson correlation analysis to assess the relationship between neighbourhood and peer attachment. The study found a negative and strong relationship (r=-.125, p-value = < 0.012) between neighbourhood and peer attachment among pregnant female students, see Table 2.

**Table 2:** Relationships Between dependents variables and Independent Variables

No	Variables	*r*	*P-Value*
1.	Neighbourhood->Peer attachment	− 0.125	< 0.012
2.	Sexual attitude->Neighbourhood	− 0.040	0.422
3.	Sexual attitude-> Academic Performance	0.236	< 0.000
4.	Peer attachment -> Academic Performance	− 0.401	< 0.001

### Relationship between neighbourhood and sexual attitudes

The second objective was to investigate the relationship between the environment and sexual attitudes. The results of the study showed that the mean rating of sexual attitudes on a scale of 1 to 10 was in the descriptive results (M = 4.70, SD = 2.21). According to the frequency data, 63% of the respondents had negative sexual attitudes while 37% had positive attitudes. Meanwhile, Pearson correlation analysis was conducted to examine the relationship between neighbourhood environment and sexual attitude. The results in Table 2 show that there is no relationship (r=-.040, p-value = 0.422) between neighbourhood and sexual attitude.

### Relationship between sexual attitude and academic performance

The descriptive results for sexual attitude showed that the mean score for sexual attitude was (M = 4.70, SD = 2.21). The frequency result also showed that 63% had poor sexual attitude and 37% had good sexual attitude. Therefore, the study concluded that the sexual attitude of the respondents was poor. Table 2 shows the result of Pearson correlation which was used to demonstrate the relationship between sexual attitude and academic performance. It showed a significant and strong positive relationship (r = .236 p-value = < 0.000).

### Relationship between peer attachment and academic performance

The fourth objective was to examine the relationship between sexual attitudes and academic performance. The results of the study showed that the mean average score for Meanwhile, Pearson correlation analysis was used to examine the relationship between attachment to peers and sexual attitudes. The results presented in Table 2 indicate a negative and moderately strong relationship (r=-.401 p-value = < 0.001) between peer attachment and academic performance.

## Discussion

Despite the numerous studies conducted on teenage pregnancies to minimise the prevalence and negative impact on the academic development of teenage girls. It is alarming to observe that the trend of this problem is constantly increasing. Numerous studies conducted on this topic have evidently failed to prevent teenage females from becoming pregnant. Therefore, it is essential to identify the factors that may hinder the academic success of adolescent students who are pregnant. Given that unintended teenage pregnancy significantly increases the school dropout rate of pregnant teenage girls in Nigeria. This prompted the present study to examine the relationship between environment (neighborhood) and individual (Sexual attitudes and peer attachment) factors and the impact of individual factors on academic performance of pregnant adolescent female students. The results indicate a significant relationship between environment and peer attachment. However, there was no significant correlation between sexual attitudes and environment. The study established statistically significant evidence between individual factors (sexual attitudes and peer attachment) and academic achievement.

This study established a relationship between environment (quality of neighbourhood) and individual factor (peer attachment (trust, communication, and alienation). In the current study, a strong and significant relationship was found between neighbourhood environment and peer attachment. This is consistent with the findings of previous studies which found that the quality of the neighbourhood predicts the nature of peer attachment. It is evident that violence, insecurity, poverty in the home and lack of access to basic human needs and health facilities often exacerbate young adults’ vulnerability to early independence and deviant behaviours [13, 50, 51]. The constant confrontation with the highlighted negative obstacles can form a socio-cultural norm of deviance for adolescents living in such environments [13, 50, 51]. Thus, young people may be unconsciously pressured to associate with unhealthy peers to be accepted in the neighbourhood. For example, Criss et al. found that an unsafe neighbourhood (danger) was a strong predictor of peer group acceptance and social behaviour in the adolescents surveyed [40]. By comparison, Haynie et al. believe that a poor neighbourhood produces prosocial adolescents, exposing them to unhealthy peer associations and negative peer influence [52]. In the current study, it was found that a significant proportion of adolescent girls lived in a poor neighbourhood where they probably lacked easy access to conducive learning environment (schools), health facilities and even recreational activities. This may have increased the risk of deviance and thus increased their vulnerability to unhealthy peer relationships. This point to the importance of safe environment in terms of access to conducive schools, health care facilities and recreational facilities within neighbourhoods. As this are identified as indicators of promoting positive peer influence and an indirect pointer to good academic assimilation and success for pregnant adolescent students in Nigeria.

Based on the premise that neighbourhood quality can influence access to and use of sexual and reproductive health services, contraceptive use, and sexual activity, as well as the ability to make informed decisions regarding unsafe sex, the current study examined the relationship between sexual attitudes and neighbourhood. This study, however, found no correlation between sexual orientation and neighbourhood. Given that a significant proportion (69.3%) of expectant respondents resided in low-quality neighbourhoods and 64.8% were from low-income families, this finding is intriguing. Invariably our finding suggests that neighbourhood factors did not predict sexual attitude, despite 63% of respondent demonstrated low ability to make informed decision regarding safe sexual practices. The result is consistent with a study by Ifegbesan et al. from Ogun State, Nigeria. Ifegbesan et al. discovered that neighbourhood quality was not a predictor of adolescents’ sexual attitudes; rather, age, communication, sex, religion, parents’ marital status, and who the adolescents resided with were superior predictors [53]. In contrast, Warner found that neighbourhood quality (High and Low end) was a predictor of teenage females’ sexual attitudes [54]. Reiterating that early sexual debut was especially prevalent in all observed neighbourhoods, regardless of quality. Nonetheless, the likelihood of early sexual début and multiple sexual partners was greater among adolescent respondents living in a low-quality neighbourhood.

Finally, this study found a correlation between individual factor (sexual attitudes) and academic integration (academic performance) among pregnant respondents. Studies suggest that adolescent’s student with history of early sexual engagement and unsafe sexual practises (non-contraceptive) are also likely to experience academic difficulties in terms of ability to focus [27, 55, 56]. This is consistent with the study by Lanari et al. which found that adolescent sexual activity is an important factor in academic performance [27]. Lanari et al. found that the more adolescents engaged in sexual activity, the worse their academic performance became. This was evident in adolescents who had little knowledge about sexual and reproductive health and contraceptive methods. The study by Whitaker and Miller[55] also mirrors the study by Lanari et al. The researchers emphasised that adolescents who are more sexually experienced and report a greater number of sexual partners are also more likey to have a greater proportion of sexually active friends, which could influence students’ academic performance [55]. According to Kumar’s study teenage girls who reportedly had good grades were void of sexual activities as compared to sexually active student who reported poor academic performance[26]. This finding mirrors that of Ventrina where adolescent female student who engagement sexual activities without use of condoms lacked the ability to balance sexual life with academic commitments[28]. This further affected their ability to concentrate and attend school regularly thereby resulting to poor academic performance[28]. A previous study by Frisco linked early sexual initiation and unprotected sex with academic disruption and diminished chances of advancing to college-level education. Frisco argued that the aforementioned factor can destabilise a student’s academic equilibrium and, in the absence of adequate support, can contribute to a low rate of school attendance and, ultimately, school dropout among sexually active and pregnant adolescents [24]. It is possible that the pregnant respondents in the current study did not have access to sexual health services and contraceptives due to poor neighbourhood conditions in their place of residence, which increased their vulnerability to unsafe sexual choices. In addition, the respondents’ pregnant status is a clear indication of an active sexual life and lack of competence to make safe sexual choices. The lack of ability to control sexual activity during pregnancy, due to the likely absence of sexual health services, as well as the reconciliation of schoolwork and pregnancy and the negative influence of peers, could therefore also be reasons for poor academic integration (performance). This substantiates the need to identify tailored social and behavioural change approaches that promote positive sexual attitudes and enhance adolescents’ decision-making abilities. This can help reduce the risk of multiple pregnancies, multiple sexual partners, and enhanced sexual autonomy in this population. Consequently, mitigating the effect of this poor sexual behaviours on academic performance.

This study assessed the relationship between peer attachment and academic achievement. Communication, trust, and alienation were used to evaluate peer attachment. Peer relationships are one of the most influential factors influencing academic achievement. This is because adolescents often seek validation and acceptance from their peers during puberty. Given this instance, it is assumed that peer attachment can play a significant role in the academic engagement and success of pregnant adolescent students. Therefore, the current study established a significant link between peer attachment and academic success. This result is consistent with that of Liu et al., as the study found that peer influence predicted student academic achievement. In other words, students who were in the company of positive influences (high commitment level with school) had higher academic achievement compared to students who were in the company of negative influences and had lower academic achievement[32]. Accordingly, Llorca et al. discovered a direct relationship between peer attachment quality and academic self-efficacy related to positive academic achievement, whereas low academic self-efficacy was directly associated with negative peers [33]. Stanard et al. found that peer relationships affected adolescents’ academic engagement in terms of emotional demands (flow in school, school burnout, and appreciation of school), cognitive demands (effort in learning), and behavior (attendance)[25]. The importance of positive peer attachment to the academic achievement of adolescent females cannot be overstated. Similarly, Jerald indicated that academic performance of teenagers can be influence by peer association. Jerald found in their study that students who identified with peers that skips school recorded poor academic success [30]. In other to improve academic achievement it is therefore of importance to enforce positive peer association among peer groups.

Lanari et al. and Parkes et al. believes that some of the keyways of reinforcing positive sexual behaviors among adolescent girls is by teaching early sexuality education[27, 56] [48].Malga et al. reiterated that positive sexual behavior can be achieved through focus on encouraging positive health and behavior change [57]. Consistently,Phongluxa et al. and Weatherley et al. proposed the implementation of life skill curriculum and comprehensive sexuality education into the national education curriculum[58, 59] [51]. Furthermore, focus on neighbourhood quality through promotion of recreational activities, access to youth friendly sexual health services, peer education to promote health and positive peer influence within the neighborhood, safe and clean neighborhood can enhance decision making competence and further improve self-efficacy, self-esteem, and confidence [31, 57, 60]. This can help minimize vulnerability towards unsafe sexual practices as well as minimize poor academic outcome and school dropout among pregnant adolescent students.

According to the findings of this study, to establish academic integration of pregnant students, it is critical to improve the individual and environmental exposure of pregnant adolescent students. As a result, the study recommends taking a prosocial and personal behaviour change approach when addressing the impact of individual factors. This could be accomplished by incorporating and enforcing sex education into school curricula, increasing the availability and affordability of contraception (subsidised or free), providing free antenatal care, peer education programmes, and providing free counselling sessions. Furthermore, pregnant students, particularly girls from socioeconomically disadvantaged families, can be supported and encouraged through the provision of free or subsidised education, which can significantly mitigate the impact of financial constraints on academic outcomes. Meanwhile, environmental reform can be established by improving access to conducive learning environment (school) and health care facilities, community programmes (vocational trainings) that can positively engage young adults in the neighbourhood, and school-based day care that can promote academic integration of pregnant students. This approach has the potential to reduce the effects of neighbourhood, negative or unhealthy peer attachment, and unsafe sexual practises on adolescent pregnancy. As a result, regardless of their pregnancy status, their self-efficacy, motivation, and reliance to achieve higher academic feats are indirectly increased.

## Conclusion

The study identifies ways to reduce the factors that may increase the likelihood of poor academic performance and school dropout among pregnant adolescent schoolgirls in Nigeria. This study found that environment (neighbourhood), sexual attitudes and peer attachment significantly predicted academic performance among pregnant female students. In addition, environment (neighbourhood) was found to correlate with peer attachment, while neighbourhood had no effect on sexual attitudes. This study demonstrated that improving the academic integration (performance) of pregnant teenagers necessitates a combination of approaches, including changes in personal behaviour, prosocial behaviour, and environmental reforms. Positive peer influence, decision-making skills related to safe sexual choices, peer education, school day-care, subsidised or contraceptives, and community programmes that positively influence young adults in the neighbourhood are all indicators. These approaches can promote healthy peer influence and sexual behaviour, reducing the impact of teenage pregnancy on pregnant adolescents’ academic success. Considering this, the study suggests that policymakers and key stakeholders should work towards establishing youth-friendly, non-discriminatory sexual, subsidised, or free education for female especially pregnant student and reproductive health services that are free or low-cost. Free counselling sessions in schools and neighbourhood school day care to care for pregnant students’ health needs. These approaches can improve academic performance while reducing school dropouts and other negative factors associated with unwanted pregnancies and low educational attainment, such as poverty, limited employment opportunities, repeated pregnancies, low bargaining power in the labour market, and so on.

### Limitation and strengths of study

Because the present study was a cross-sectional study with a quantitative design, it may not have been possible to demonstrate the cause-and-effect relationships of the variables studied. A longitudinal study might be more effective in examining the relationship. In addition, we only studied pregnant adolescent female students who were present and did not include pregnant female students who dropped out of school. A comparison between students and dropouts could have helped inform recommendations that would help dropouts return to school. Regardless, the current study identified respondents from different geopolitical regions that are representative of the country, which strengthens recommendations.

## Data Availability

The datasets used and/or analysed during the current study available from the corresponding author on reasonable request.
